# Buffalo Medical and Surgical Association

**Published:** 1885-12

**Authors:** 


### BUFFALO MEDICAL AND SURGICAL ASSOCIATION.

*Meeting of Nov. 10, 1885.*

The Vice-President, Dr. W. W. Potter, in the chair; Dr. F. R. Campbell, Secretary. Present—Drs. Briggs, Brecht, Crego, Bartow, Coakley, Hayd, Howell, Howe, Chas. Ring, Grosvenor and Broardt.

The reading of the minutes of the last meeting was postponed for one month, as the Secretary had failed to bring them to the meeting.

The name of Dr. Norton was proposed for membership.

Dr. F. R. Campbell, the essayist of the evening, read a paper, entitled, "The Relation of Meteorology to Disease." (Published in this number of the JOURNAL.)

*Discussion.*—Dr. Briggs spoke of the importance of such papers as the one presented to the Association, for pointing out the causes of epidemics and preventible diseases. He well remembered the epidemic dysentery of 1881, and called attention to the fact that it was most prevalent in the fifth and sixth wards, where well water was then generally used and the sewer system was very imperfect. Dr. Briggs called attention to the importance of keeping a careful record of vital statistics, and requested the profession of the city, through the Association, to exercise more care in filling out death certificates. Symptoms of diseases, such as dropsy, convulsions, and even "fitz," were sometimes given as the cause of death. He called attention to a city ordinance which directed all physicians meeting with cases of contagious disease, such as diphtheria, scarlet fever, typhoid fever, small-pox, measles, etc., to report these cases to the office of the Board of Health within twenty-four hours, or be liable to a fine of fifty dollars for each offense. When cases of disease, and not merely the deaths, will be recorded at the Health Office, the study of the subjects connected with preventive medicine, will be greatly facilitated. Attention was called to the influence of locality on the death rate. The healthiest ward in Buffalo is the Ninth, and next to it stands the "infected" Eighth, which has a low death rate in spite of its bad sanitary condition. The most unhealthy wards were the Fifth and Sixth.

Dr. Grosvenor considered the subject one of great practical utility. By knowing what seasons and conditions of the weather were most injurious to patients with certain diseases, we can teach them how to avoid their evil effects. By knowing what meteorological conditions were most favorable to a given disease, we could make use of our knowledge in treatment, sending our patients to a climate most favorable to recovery.

Dr. Coakley had observed the changes of diseases with the seasons, but had never made a study of the subject. He did not believe that the weight of the body increased in summer in this country, but was convinced, from common experience, that quite the opposite occurred. Thought that weight diminished in the hot season here, because evaporation was greater in summer in our climate than in winter.

Dr. Crego did not agree with the conclusion of the essayist regarding our winter being more favorable for consumptives than summer. It was common belief in the profession, and it was in accord with his experience, that a high altitude with low humidity was the best resort. He had seen very bad results among patients and friends from a trip to the south during winter; hence, to compare Buffalo and the south, and draw conclusions, was manifestly not correct. He had read with interest, very lately, Dr. Weir Mitchell's article referred to by Dr. Campbell—the relation of pain to weather. He had also seen patients who could predict the coming of a storm as accurately as the Signal Service Department. He called attention to the nervous symptoms with which some patients suffered at the approach of a storm, and that certain states of the atmosphere aggravate the symptoms in many forms of nervous troubles. It had also occurred to him that certain mental and nervous diseases were developed during certain seasons, while another class was developed at another time. This statement he was, of course, unable at present to verify.

Dr. Howe called attention to the fact, in substantiation of Dr. Crego's statement, that he knew of a patient who had a slight attack of melancholia every year, at a certain season, and that it passed off when the weather changed. It is a well-known fact that certain forms of eye trouble depended on the state of the weather; that conjunctivitis was in the city, for instance, confined almost entirely to certain seasons.

Dr. W. W. Potter called attention to the vast amount of labor required to collect and formulate statistics for such a paper as the one under discussion. Some of the inferences drawn were in direct opposition to his preconceived notions; but he thought that a scientific study of such a subject as this would overturn many other common beliefs.

Dr. Campbell, in conclusion, pointed out the fact that the action of meteorological agents, in the production of disease, was principally indirect, and the objects through which they act are often under man's control. Were it not for this, the study of medical meteorology would have no connection with preventive medicine. In regard to Dr. Coakley's theory of the increase of the weight of the human body in summer, he stated that the relative humidity of the air in England was not above that of this city, and thought the supposed increase of weight was due to the heavier wearing apparel worn in winter. In regard to the best climate for consumption, he considered dryness and low range of temperature most important. The higher the atmospheric pressure, the better. The only reason for sending a patient to a high altitude was, that the air in such places was dry. The most dangerous season for consumptives in Western New York is the spring; the most dangerous period for consumptives in the south is winter, which resembles our spring.

*Voluntary Communications.*—Dr. Hayd presented a typical case of *tinea favosa*, which, he stated, was very rare in this country, but was more common in England. The patient was a boot-black, and had had the disease from early childhood; some of the other members of the family were afflicted with the same disease.

Dr. Howe presented a pathological specimen, the temporal bone of a patient who had chronic otorrhea with necrosis of bone. The patient died of acute meningitis, produced by the diseased bone.

*Miscellaneous Business.*—Dr. W. W. Potter read a communication from the New York Physicians' Mutual Aid Association, making inquiries regarding the advisability of extending the membership of the Association in Buffalo.

Dr. Briggs mentioned the fact that there was a Physicians' Mutual Aid Association in Buffalo. The fund derived from the initiation fees was deposited in the Erie County Savings Bank. No assessments had been made, no dues had been paid, and the first member who died would break the institution.

The meeting then adjourned.

